# Comparative genomic analyses of freshly isolated *Giardia intestinalis* assemblage A isolates

**DOI:** 10.1186/s12864-015-1893-6

**Published:** 2015-09-15

**Authors:** Johan Ankarklev, Oscar Franzén, Dimitra Peirasmaki, Jon Jerlström-Hultqvist, Marianne Lebbad, Jan Andersson, Björn Andersson, Staffan G. Svärd

**Affiliations:** Department of Cell and Molecular Biology, Science for Life Laboratory, BMC, Uppsala University, Box 596, SE-751 24 Uppsala, Sweden; Department of Cell and Molecular Biology, Karolinska Institutet, Box 285, SE-171 77 Stockholm, Sweden; Science for Life Laboratory, KISP, Tomtebodavägen 23A, 171 65 Solna, Sweden; Department of Microbiology, Public Health Agency of Sweden, SE-171 82 Solna, Sweden

## Abstract

**Background:**

The diarrhea-causing protozoan *Giardia intestinalis* makes up a species complex of eight different assemblages (A-H), where assemblage A and B infect humans. Comparative whole-genome analyses of three of these assemblages have shown that there is significant divergence at the inter-assemblage level, however little is currently known regarding variation at the intra-assemblage level. We have performed whole genome sequencing of two sub-assemblage AII isolates, recently axenized from symptomatic human patients, to study the biological and genetic diversity within assemblage A isolates.

**Results:**

Several biological differences between the new and earlier characterized assemblage A isolates were identified, including a difference in growth medium preference. The two AII isolates were of different sub-assemblage types (AII-1 [AS175] and AII-2 [AS98]) and showed size differences in the smallest chromosomes. The amount of genetic diversity was characterized in relation to the genome of the *Giardia* reference isolate WB, an assemblage AI isolate. Our analyses indicate that the divergence between AI and AII is approximately 1 %, represented by ~100,000 single nucleotide polymorphisms (SNP) distributed over the chromosomes with enrichment in variable genomic regions containing surface antigens. The level of allelic sequence heterozygosity (ASH) in the two AII isolates was found to be 0.25–0.35 %, which is 25–30 fold higher than in the WB isolate and 10 fold higher than the assemblage AII isolate DH (0.037 %). 35 protein-encoding genes, not found in the WB genome, were identified in the two AII genomes. The large gene families of variant-specific surface proteins (VSPs) and high cysteine membrane proteins (HCMPs) showed isolate-specific divergences of the gene repertoires. Certain genes, often in small gene families with 2 to 8 members, localize to the variable regions of the genomes and show high sequence diversity between the assemblage A isolates. One of the families, Bactericidal/Permeability Increasing-like protein (BPIL), with eight members was characterized further and the proteins were shown to localize to the ER in trophozoites.

**Conclusions:**

*Giardia* genomes are modular with highly conserved core regions mixed up by variable regions containing high levels of ASH, SNPs and variable surface antigens. There are significant genomic variations in assemblage A isolates, in terms of chromosome size, gene content, surface protein repertoire and gene polymorphisms and these differences mainly localize to the variable regions of the genomes. The large genetic differences within one assemblage of *G. intestinalis* strengthen the argument that the assemblages represent different *Giardia* species.

**Electronic supplementary material:**

The online version of this article (doi:10.1186/s12864-015-1893-6) contains supplementary material, which is available to authorized users.

## Background

*Giardia intestinalis* (syn. *Giardia lamblia*, *Giardia duodenalis*) is an intestinal protozoan parasite that colonizes the small intestine of humans and other mammals and it is a major cause of diarrhea worldwide. Endemic outbreaks are common in low-resource settings, where infections are linked to malnutrition, mortality and growth retardation in children [1]. Symptoms associated with the infection vary, from asymptomatic to severe, where diarrhea encompassed by vomiting, bloating, nausea and fatigue are common traits, and immunocompromised individuals are at higher risk of infection as well as clinical manifestation [2].

*G. intestinalis* is divided into eight morphologically identical genotypes or assemblages (A to H). However, only A and B have been associated with human infections. Assemblage A and B are further divided into sub-assemblages: AI, AII, AIII, BIII, and BIV [3]. Despite extensive efforts to associate specific assemblages to symptoms, conflicting results have been obtained and there is to date no clear correlation between assemblage and symptoms. However, it has been reported that genotype AI is more frequently found in animals, whereas genotype AII is mainly found in humans [3, 4]. Certain assemblage A isolates have been connected to symptomatic *Giardia* infections in humans [5] and some have been suggested to have zoonotic potential [6, 7]. However, most typing studies have used a limited panel of genes with low substitution rates, mainly due to the lack of genome data from other assemblages or subgroups.

The genomes of three isolates from the two human infecting assemblages (AI, AII and BIV) have been sequenced and analyzed [8–10] along that of the hoofed-animal infecting assemblage E [11]. The genome of *G. intestinalis* consists of 5 chromosomes, and sequencing efforts have shown the genome to be compact in terms of gene content and size; the haploid genome is ~10.7–12 Mbp, with relatively little non-coding sequence compared to most eukaryotes [10]. Untranslated regions of mRNAs are relatively short [12, 13] and only a few genes have been shown to contain introns [1]. The Variant-specific Surface Proteins (VSP) and the High Cysteine Membrane Proteins (HCMP) are two major, highly-variable, multi-gene families that are found in the genomes of *G. intestinalis* and are associated with antigenic variation and immune evasion [1, 10].

Despite the relatively large divergence between assemblages A, B and E, comparative genomics have identified a conserved core of protein encoding genes (~4500) [11]. Analysis of the assemblage B genome (isolate GS) showed extensive allelic sequence heterozygosity (ASH) within the genome [9], ASH was lower in the AII isolate DH (ASH 0.037 %, [10]) whereas it was even lower in assemblage AI (isolate WB; ASH <0.01 %) [8] and E (isolate P15; ASH ~0.0023 %) [11]. Several bacterial-like assemblage-specific genes have been identified in the sequenced genomes, which are likely due to reflect recent lateral transfers from gut bacteria [8, 9, 11].

The genome efforts that have been performed to date have increased the understanding of the genetic landscape of *G. intestinalis*, but the extent of diversity within each assemblage is less explored, and the chromosome-wide distribution of genetic diversity is still poorly defined. Broadening the knowledge regarding genome-wide genetic diversity in pathogen biology is important in several aspects as it enables improvements in genotyping strategies, diagnostics and molecular epidemiology, and may reveal signatures that can relate to infectivity, pathogenesis and drug resistance. We have performed whole genome sequencing of two recently axenized *G. intestinalis* assemblage AII isolates with confirmed pathogenicity in humans, using the chromosome-level assembly of the *G. intestinalis* assemblage AI (WB) genome sequence as reference, with the aim to characterize the amount of genetic diversity between the two human infecting sub-assemblages AI and AII.

## Results

### Clinical and biological data

Two new assemblage AII isolates were individually isolated from two female patients in Sweden that were infected in Sweden (AS175) and India (AS98), respectively. The patients suffered from a wide range of typical giardiasis symptoms including; watery diarrhea, vomiting, nausea, fatigue, and weight loss. Initial genotyping of *Giardia* cyst DNA extracted from the infected patient fecal samples using beta-giardin (*bg*), triose-phosphate isomerase (*tpi*) and glutamate dehydrogenase (*gdh*) typing showed that AS98 had a multi-locus genotype (MLG) corresponding to MLG AII-2 [7] and AS175 had the AII-1 MLG. Typing using assemblage-specific *tpi* primers [14] showed small traces of assemblage B DNA in the AS98 sample whereas the AS175 sample only contained assemblage A DNA (data not shown). MLG analysis of the two isolates after growth *in vitro* (10 passages) showed the same MLG genotype (AII-1) of the AS175 isolate and exclusively MLG AII-2 in the AS98 isolate.

The two isolates were axenized after *in vitro* excystation and several biological differences were identified when the isolates were grown *in vitro*. Interestingly, the two new AII isolates grew faster in medium containing human serum compared to the standard medium with bovine serum where no or little growth was seen (Additional file 1). The growth rate of AS98 at optimal conditions was similar to the WB (AI) isolate whereas AS175 showed a slightly slower growth rate (Additional file 1). The encystation efficiency of the AII isolates was comparable to the WB isolate, which is known as one of the best encysting isolates *in vitro* [1]. Pulsed field electrophoresis analyses of the highly characterized assemblage AI isolate WB, compared to AS98 and AS175, showed size differences in the smallest chromosomes (Chromosome 1 and 2; Additional file 2). In WB, chromosomes 1 and 2 have the same size (1.55 Mbp) whereas the smallest chromosomes in AS98 are 1.70 Mbp and 1.55 and 1.70 Mbp in the AS175 isolate. Similar differences in chromosome 1 and 2 sizes between assemblage A isolates have previously been noticed and it was suggested to be due to recombination between telomeric rDNA genes [15, 16]. Our sequencing data could not reveal the reason to the differences in chromosome size between the three A isolates but further analyses using long-read sequencing techniques like PacBio and optical mapping can resolve this issue.

### Genome assembly and isolate specific sequences

Total DNA was extracted from the two AII isolates after minimal growth time *in vitro* (10 passages). This was done in order to reduce the risk for genomic changes due to *in vitro* growth. Genomic DNAs from the two isolates were individually sequenced using 454-sequencing (see Methods). The average sequence coverage of the final assemblies were 8X (AS98) and 30X (AS175), see Additional file 3. The sequenced AII genomes were found to be sufficiently similar to the available genome sequence of assemblage AI (isolate WB) to allow a reference-guided mapping of the reads, using the chromosome-level assembly of isolate WB as a template. Reads with more than one mapping location were removed to avoid bias caused by multi-mapping reads. The contig N50s were 40 and 57 kb for AS98 and AS175, respectively. The number of sequence gaps was similar in both assemblies, and were on average 660 bp/gap, corresponding to 931,756 and 814,954 unmapped positions or about 88–9 % of the genome. Regions that were not possible to cover, i.e. the gaps, were found to correlate to the more flexible part of the genome, which we have previously estimated to approximately 9 % of the genome [11]. The lower sequencing output of the AS98 genome caused a slightly higher gap count in this assembly.

The core genomes of the three isolates were very similar and most sequence reads from the two AII genomes aligned well with the AI reference genome. A small number of high quality reads (n_AS98_ = 5468, n_AS175_ = 9018) did not align with the WB genome, and these were therefore assembled *de novo* (see Methods), which resulted in a small number of short contigs (n_AS98_ = 111, n_AS175_ = 94; N50_AS98_ = 1467 bp, N50_AS175_ = 2098 bp). The gene content of these was analyzed, as these could contain isolate-specific sequences that are absent from the reference genome. Among the identified genes were one REP2 viral-like replication protein, one DNA polymerase and several hypothetical proteins with similarity to genes in the GS genome [9]. In total, 35 genes were found to be present in the two AII isolates but absent in the AI genome (Additional file 4). One 1109 aa hypothetical WB protein (GL50803_137673) was found missing in AS175, but a diverged homolog was found in the recently sequenced AII isolate DH and in AS98 on one of the *de novo* assembled contigs. Furthermore, one WB NEK Kinase (GL50803_9327) and one gene encoding an ankyrin-repeat protein (GL50803_14926) were found to lack syntenic orthologs in AS98 and DH but not in AS175. One WB ankyrin repeat protein (GL50803_9605), a NEK Kinase (GL50803_101534) and one hypothetical 251 aa protein (GL50803_137678) were found to be missing in all three AII isolates. This data show that there are sub-assemblage specific proteins in *Giardia* that potentially can be used for genotyping.

The coverage of each chromosome was inspected both manually and using automatic search tools, in order to identify putative segmental duplications or evidence of aneuploidy since chromosome size differences were detected in the pulse-field analysis. Despite local variation in read depth, the distribution and size of the coverage peaks did not suggest any large structural duplications or aneuploidy (see Methods). Most read depth variation was attributed to artifacts related to the emulsion PCR, rather than actual genomic differences. However, certain regions appear with higher than average read depth, and with reads having different orientation and start/stop positions, in relation to what can be expected by artifacts alone. This likely indicates differences in gene copy number in the AII genomes. Interestingly, most of these genes are typically found in the telomeric regions [16]. In AS175, genes with higher than average depth include; reverse transcriptase elements, VSP genes, ribosomal RNA genes and one gene encoding the axoneme-associated protein GASP-180, one multidrug resistance-associated protein 1, proprotein convertase subtilisin/kexin type 5 precursor. In AS98, similarly reverse transcriptase genes were found to have larger than average read depth, and one gene encoding an ABC transporter and a multidrug resistance-associated protein. Overall, these analyses support the earlier observation of chromosome size differences due to translocations in the telomeric regions.

### Analysis of multi-gene families

**Fig. 1:**
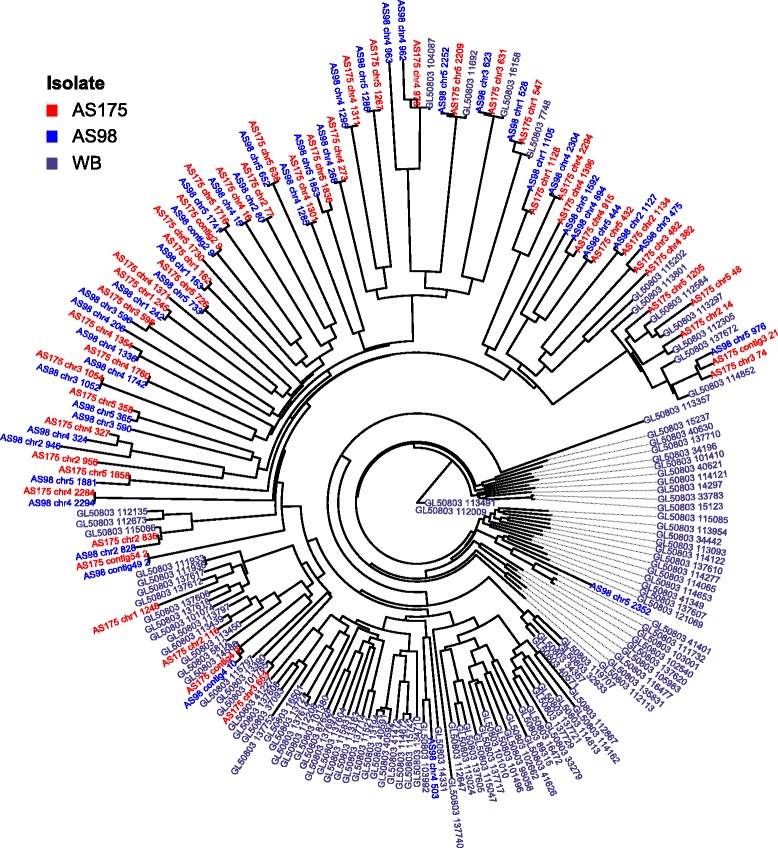
Analysis of VSP genes. VSP diversity in *Giardia intestinalis* assemblage A isolates. Black: WB (AI), Blue: AS98 (AII-1) and Red: AS175 (AII-2). An alignment-free method was used to compare the VSP repertoires. The VSPs from the three different assemblage A isolates form two major cluster with the AI VSPs separated from the AII VSPs

Variant specific surface proteins (VSPs) is a multi-gene family in *Giardia* that is notoriously difficult to study, both using sequencing and by functional investigation [17]. The genome sequencing approach used here cannot completely reconstruct the genomic repertoire of these genes, neither using a reference assembly nor a *de novo* assembly strategy. To overcome this, we have quantified the relative amount of VSP-like sequences using a recently published alignment-free method [18], see Methods. This analysis shows that the VSP repertories of assemblage A isolates can be divided into two main clusters with the WB (AI) VSPs in one cluster and the VSPs from the AII isolates in the other (Fig. 1). Within these clusters it is apparent that there are a few conserved VSPs found in all three assemblage A genomes (Fig. 1) but the majority of the VSP repertoires have evolved independently in the studied assemblage AI and AII isolates (Fig. 1). A similar pattern was obvious in the smaller (around 60 genes) and less variable HCMP gene family [19] when compared using MEME analysis (Additional file 5). Several HCMPs are conserved between the isolates but isolate specific divergences have occurred. Thus, the main mechanism of expansion in these two multi-gene families seem to be isolate-specific duplications of certain VSPs and HCMPs followed by divergence of the duplicated genes.

### Allelic sequence heterozygosity

The AS98 and AS175 assemblies contained an abundance of high quality mismatches between aligned reads. This has been identified in earlier *Giardia* genome sequencing projects [9–11] and the mismatches represent heterozygous bases due to sequence differences between the four different genomic copies of the *Giardia* genome, distributed in two nuclei [20]. In *Giardia* this type of genomic variability has been named allelic sequence heterozygosity (ASH) [21]. The amount of such putative ASH in the AS98 and AS175 genomes was estimated by counting the number of high quality mismatches between aligned reads, as outlined in Methods. This search identified a total of 26,148 heterozygotic positions in AS98 and 22,661 in AS175, which yields an average percentage of heterozygosity 0.35 % ASH for AS98 and 0.25 % for AS175. This represents a 25–35 fold higher heterozygosity level than what was found in isolate WB (AI) and P15 (E; [11]), 10 fold higher than the DH isolate (AII, [10]) but slightly less than what was found in GS (BIV, 0.425-0.53 %; [9]).

The most common type of heterozygosity was found to contain two different bases. Heterozygotic positions with three different bases were found only in 1,920 and 4,585 positions (AS98 and AS175, respectively) and less than 150 positions had four different bases. About 80 % of the observed heterozygotic positions were transitions, consistent with the theory of nucleotide substitutions. In total, about 30 % of the heterozygosity was located in the core gene content, whereas the rest was present in either non-core genes such as VSPs and HCMPs or in non-coding sequence. However, since heterozygosity was also identified in core genes, this represents variation that potentially can give rise to an additional number of different protein isoforms, either at the population level or at the single cell level.

**Fig. 2:**
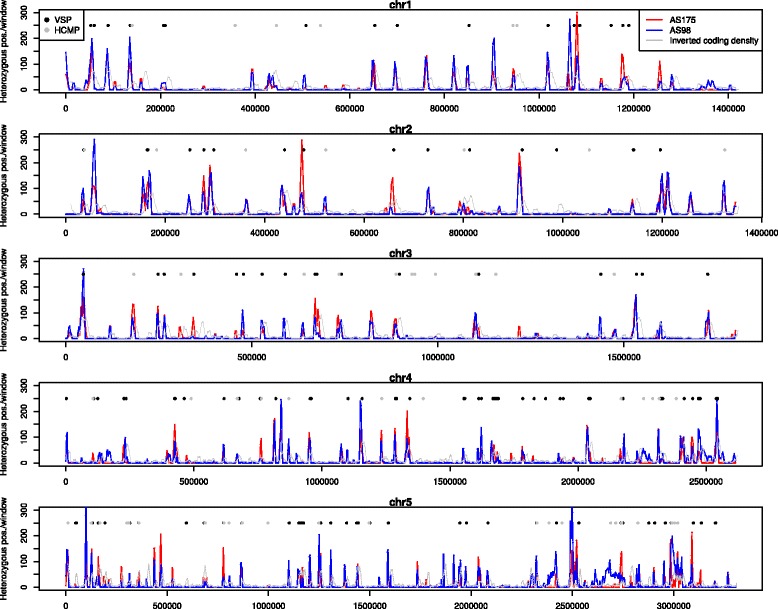
Putative allelic sequence heterozygosity. Chromosome-wide distribution of putative allelic sequence heterozygosity (ASH). Red lines represent ASH in isolate AS175 (assemblage AII-1) and blue lines represent ASH in isolate AS98 (assemblage AII-2). Grey lines represents the inverted coding density, ie. more intense peaks represent less coding content and lower peaks represent higher coding content. Dots indicate the approximate chromosomal location of VSP genes (black) and HCMP genes (grey). Regions with higher content of ASH coincide with regions of lower coding content and often a VSP or HCMP gene. Regions with high coding content tend to be more devoid of ASH

**Fig. 3:**
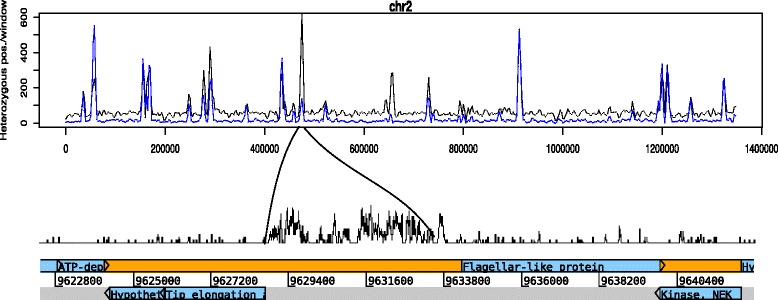
Close-up of ASH in a specific intergenic region on chromosome 2. The blue line represents ASH in isolate AS175 (assemblage AII-1) and grey line represents ASH in isolate AS98 (assemblage AII-2)

Chromosomes-wide distribution plots indicated heterozygosity not to be homogeneous, rather high-density regions are intervened with regions devoid of ASH (Fig. 2), a pattern also observed in the GS (assemblage B) genome [9]. Regions with a lower proportion of coding content coincide with regions with a high degree of ASH and the presence of VSP and HCMP genes (Fig. 2). Higher coding content was in most cases found to correlate with the absence of, or very low levels of ASH, as expected by the selective pressure in these regions, which allows them to accumulate less variation. The plots for the two AII genomes display almost over-lapping curves (Fig. 2), indicating that regions prone to ASH accumulation are similar, at least in these AII genomes. However, the intensity of peaks can differ even within the AII group, possibly reflecting local variation in ASH accumulation (Fig. 3). In most cases AS98 have more intense peaks, but there are several exceptions where the peaks are more profound in AS175 (Fig. 3). The chromosome-wide plots further reveal that each chromosome has on average 20–30 regions with extensive ASH accumulation. In *Giardia*, chromosome 5 is the largest (~3.3 Mbp) and is more than 2 fold the size of chromosome 1, and it also contains the largest amount of heterozygosity. Furthermore, genomic regions longer than 100 kb are devoid of ASH, rendering them as possible selective sweeps.

### Genome-wide patterns of single nucleotide polymorphisms

**Fig. 4:**
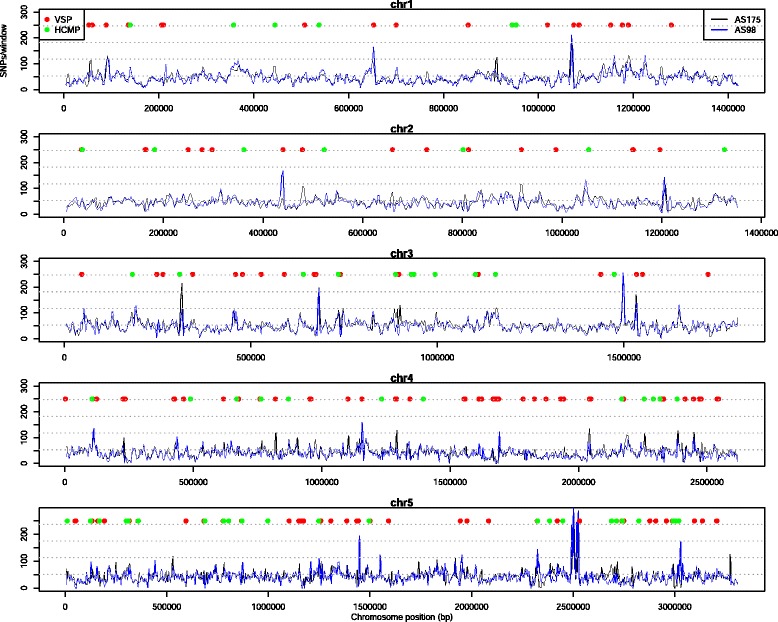
Chromosome-wide distribution of single nucleotide polymorphisms. Chromosome-wide distribution of single nucleotide polymorphisms in 15 kb overlaping windows for each of the two isolates compared to WB. The blue lines represents the AS98 isolate compared to WB and the black lines represent AS175 compared to WB. The number of SNPs is shown on the y-axis and the chromosomal position on the x-axis

We analyzed the patterns of single nucleotide polymorphisms (SNPs) along the five *Giardia* chromosomes plus several unassigned contigs using an in-house pipeline and a sliding window analysis, as shown in Fig. 4. The number of SNPs per sliding window were counted and plotted along the chromosomes, to identify regional variation in SNP rates. As expected, both AII genomes resemble each other in terms of SNPs to AI. However, the AII genomes contain areas with divergence between each other, indicating sequence diversity on the sub-assemblage level (Fig. 4). Furthermore, between AI and AII, several regions on each chromosome have an elevated number of SNPs. Frequently these regions were found to correlate with the location of VSPs or HCMPs and intergenic regions.

In total 93,900 and 100,273 SNPs were identified in the comparisons to the WB isolate, see Table 1. Thus, between assemblage AI and AII there were on average 8–9 SNPs per 1000 bp. Hence, the average sequence identity across AI and AII is >99 %, confirming previous estimations [22]. However, despite the fact that the number of identified SNPs compared to WB is similar for AS98 and AS175, the AII isolates only share about 57,680 SNPs, indicating diversity within the AII group and that population studies with new genotyping tools can reveal further substructure within this groups. The vast majority (~90 %) of the identified SNPs were located in coding sequences, as expected due to the high coding density in *Giardia*. About 32 % of the coding SNPs were found to be non-synonymous, and of these about 29 % were non-conservative amino acid changes dispersed in ~2,500 different genes.

**Table 1:** Summary of sequenced data and diversity between assemblage AI and AII

Chr^a^	% Sequence coverage^b^		% Mapped data^c^		# Coding SNPs		# Noncoding SNPs	
	AS175	AS98	AS175	AS98	AS175	AS98	AS175	AS98
1	95	95	13	13	11298	10792	2352	2146
2	95	94	12	11	10734	10240	1866	1763
3	96	94	16	15	15810	15041	3121	2740
4	96	95	22	23	18949	17298	3311	2928
5	94	93	29	28	24545	23593	4413	4163
unassigned contigs			4	4	2288	1982	1586	1214
Total	80	79	96	94	83624	78946	16649	14954

^a^Chromosome number

^b^Percentage sequenced of each chromosome

^c^Percentage of the total sequence data for each isolate that aligned to each chromosome respectively

To examine if any particular gene was undergoing positive selection, we searched for ortholog pairs between AI and AII with a dN/dS (ω) > 1. As expected, ω values were left skewed, indicating that most genes were under purifying selection. Some 64 ortholog pairs were shown to have ω >1, but the divergence was not found to be sufficient to determine if this is due to positive selection or drift. Moreover, the average ω per chromosome was investigated, which showed chromosome 1, 2 and 3 to have almost identical values (ω_chr1_ = 0.21, ω_chr2_ = 0.21, ω_chr3_ = 0.22), but slightly elevated for 4 and 5 (ω_chr4_ = 0.25, ω_chr5_ = 0.24). This is possibly caused by the higher number of surface antigen genes that reside on these chromosomes as compared to the smaller chromosomes 1, 2 and 3.

Further, we examined the nucleotide diversity between assemblage AI and AII within protein encoding regions and a complete list of ortholog pairs and the calculated nucleotide diversity is provided in Additional file 6. Molecular studies of clinical isolates have suggested that there are differences in virulence and zoonotic potential between different *Giardia* assemblages [5, 7, 23]. Assemblage A has been shown to be associated with zoonotic transmission [3, 7] and with acute diarrhea in humans [5, 7]. Differences in symptoms can be due to genetic differences in virulence factors so we investigated if any of the genes displaying high nucleotide diversity could be connected to virulence. Several proteins with high nucleotide diversity are small (94-205aa) and specific for assemblage A. Many have putative signal sequences and/or trans-membrane regions (e.g., WB ORFs: GL50803_2710, 5629, 11050, 14917, 19223, 23808, and 98122) and they localize in the same regions as variable genes like VSPs, HCMPs and NEK kinases. NADPH oxidoreductase (GL50803_ 15004) is involved in protection against reactive oxygen species in *Giardia* [24]. Over-expression of the enzyme in *Giardia* reduces the sensitivity against oxygen [24]. The enzyme is also encoded by two other genes (GL50803_ 17150 and 17151) and ORF 17150 also shows a high grade of nucleotide diversity between the assemblage A isolates and is up-regulated during host cell-interactions [25]. Several HCMP proteins are up-regulated during host-parasite interactions *in vitro* and the three most highly up-regulated genes HCMP in WB (GL50803_ 7715, 15521, and 91707 [25]) are among the genes with high level of nucleotide diversity within assemblage A. More detailed investigation of the genes and gene-families outlined above will likely provide new and important information that will aid in broadening the knowledge of virulence and potentially host specificity in *G intestinalis*.

### Characterization of the Bactericidal Permeability Increasing (BPI) protein family

**Fig. 5:**
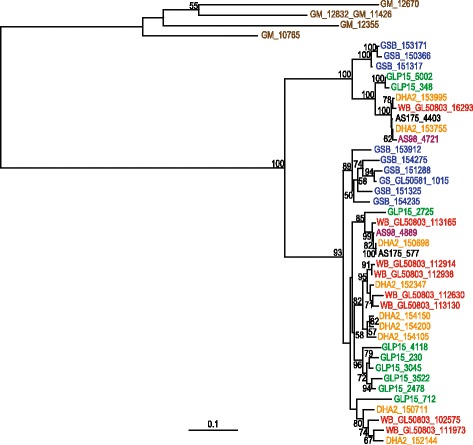
Phylogenetic analysis of Bacteriocidal and Permeability Inducing-like (BPIL) proteins in *Giardia.* Amino acid maximum likelihood phylogeny based on 485 unambiguously aligned amino acid positions. The sequences are color coded according to isolates: *G. intestinalis* WB (red), *G. intestinalis* DH (orange), *G. intestinalis* GS (blue), *G. intestinalis* P15 (green), and *G. muris* (brown). Bootstrap support values >50 are shown. The tree is rooted on the branch leading to *G. muris*

**Fig. 6:**
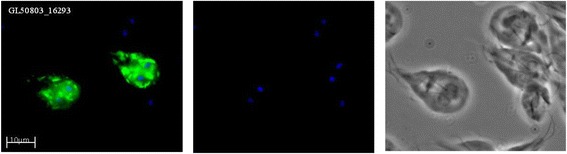
Localization of BPI-like proteins in *Giardia*. The BPI-like protein GL50803_16293 was epitope-tagged using a triple HA-tag and the fusion protein was localized to the ER in *Giardia* trophozoites using an anti-HA antibody

Two WB proteins in the top 30 list of genes with high nucleotide diversity (GL50803_16293 and GL50803_113165) are part of a small family of genes encoding proteins similar to Bactericidal Permeability Increasing protein (BPI, Additional file 6). BPI proteins are highly expressed in neutrophils and they have antibacterial activity, mainly against Gram negatives due to their LPS binding activity [26]. The *Giardia* BPI-like proteins (BPIL) are 503 aa proteins with signal peptides and structure predictions suggest that they fold into the typical boomerang-shape of BPI (Additional file 7). A phylogenetic analysis of the BPIL proteins in the *Giardia* isolates WB (8 genes), GS (8 genes), DH (9 genes), P15 (9 genes) and the mouse parasite *Giardia muris* (5 genes) showed that the genes have evolved independently in the different species/assemblages (Fig. 5). However, there are two main clusters with the two quickly diverging WB BPILs in each cluster (Fig. 5). The BPIL genes localize to flexible regions of the genome that contain VSPs, HCMPs and NEK kinases, which can explain the high level of sequence divergence. Expression analyses using RNA sequencing data showed that all genes are expressed in trophozoites of the WB, GS, P15 and AS175 isolates grown *in vitro* [12]. Epitope tagging and immunolocalization of the BPIL proteins in the WB isolate showed that they all localize to the ER (Fig. 6 and Additional file 8). Further qualitative studies are necessary in order to determine the exact function of the BPIL protein family in *Giardia*. From a microbial ecology standpoint it would be intriguing if the BPILs share similarity with the BPIs found in neutrophils, as this would give *Giardia* a competitive niche in the intestine of its host, and could potentially provide new avenues of research on the topic of microbial interactions.

## Discussion

In this study we axenized two new *G. intestinalis* assemblage A isolates (AS98 and AS175) from symptomatic human patients, performed a set of comparative phenotypic analyses, sequenced and compared their genomes. Axenization of *G. intestinalis* from patient stool samples is known to be a difficult task, however certain assemblages and sub-assemblages appear to be better adapted to currently published protocols. In our hands, the establishment of AII isolates *in vitro*, was dependent on supplementing 10 % human serum to the growth medium instead of the usual 10 % bovine serum. This indicates that species-specific serum factors may be necessary in order to establish *in vitro* growth of certain *Giardia* isolates that are more host-adapted. The use of serum from the particular host where the parasites are isolated could be a relatively simple method to improve the frequency of axenization. The two, freshly axenized, *G. intestinalis* AII isolates showed similar *in vitro* growth rates as the reference WB (AI) isolate, after minimal adaptation to *in vitro* conditions, indicating that assemblage A may generally be more easily adaptable compared to the other human infecting assemblage B, which has proven difficult to adapt *in vitro* [27]. Furthermore, after successful axenization of the AII strains, they also showed similar *in vitro* encystation rates as WB. Efficient encystation of two other anexic assemblage AI isolates was recently shown [28] but to date, efficient *in vitro* encystation of assemblage B parasites has not been possible in standard encystation medium [29], further highlightning the phenotypic differences found between *G. intestinalis* assemblages and sub-assemblages. Improved strategies to further evaluate the impact of modified culture conditions upon *in vitro* excystation and axenization, would be highly informative in light of elucidating the range of host specificity among the *G. intestinalis* assemblages and sub-assemblages.

Comparison of chromosome size between the two clinical assemblage AII strains and the reference strain WB (AI), indicate difference in size of the small chromosomes, 1 and 2. Previous studies have suggested these differences to be due to recombination of regions of rDNA in the telomeres [30]. Many pathogenic protozoan parasites, e.g. *Plasmodium falciparum* and *Trypanosoma brucei* have high densities of variable surface proteins in the telomeric regions [31, 32]. The telomeric location is important for regulation of gene-expression and in the generation of new gene variants via recombination and gene conversion [31, 32]. The WB isolate have variable surface proteins spread over the chromosomes but the density is higher close to the telomeres [8]. However, not much is known about the regulation of these genes since the so far sequenced *Giardia* genomes lack complete telomeric regions. Further genome characterizations of *Giardia* isolates using techniques that can generate assemblies of full length chromosomes, including the telomeric regions, will make it possible to study if chromosome size differences is also related to VSP expression and/or pathogenesis.

We studied the genome-wide variations between *G. intestinalis* sub-assemblage AI and AII and found that the two sub-assemblages are 99 % identical, or expressed in number of nucleotide differences, 8 SNPs per 1000 bp or around 100,000 SNPs in total. This gives clear evidence that AI and AII represent two distinct evolutionary lineages of *G. intestinalis* with several AI- and AII-specific gene variants that can potentially reflect local adaptations. From multi-locus genotyping of clinical isolates it has been suggested that AI is a more homogeneous group as compared to AII [3]. Recent proteomic analyses of two AI isolates from Australia, B-2014 and H-106, using the WB genome as reference confirmed the low level of divergence in the AI sub-assemblage [33]. This is also supported by results from genomic comparisons between the two AI isolates WB and Portland-1 ([34], 7.5 SNPs per 100.000) combined with our findings of genetic polymorphisms between AS98 and AS175 (350 SNPs per 100 000). We found that nucleotide differences or SNPs are dispersed along the chromosomes, but with local fluctuations in SNP density, reflecting either strong selective forces or the lack thereof. Dense SNP regions were frequently found in non-coding regions or in regions with variable surface proteins, suggesting that random mutations accumulate in these regions.

The level of ASH in the AII isolates (0.25–0.35 %) is similar to the ASH in assemblage B isolate GS (0.5 %) [11]. This was unexpected, since it has previously been reported that assemblage A isolates have a low level of ASH (WB <0.01 % and DH 0.037 %) [8]. Genetic exchange in *Giardia* has been extensively discussed, but to date there is no direct evidence for this process, although some reports have suggested that recombination might occur between *Giardia* isolates [35–39]. The degree of genetic heterozygosity (SNPs and ASH) in any to date sequenced *Giardia* isolate do not support the idea of frequent large scale genetic exchange in this organism, especially not between assemblages [40]. Multi-locus typing based on a limited number of genes is the most common method to discriminate between *Giardia* assemblages [4]. In our earlier studies using multi-locus genotyping with three genetic markers on 192 human *Giardia* isolates from Swedish giardiasis patients we noticed that different alleles are combined into different combinations in the different assemblage B isolates [7], suggesting recombination between assemblage B isolates. In order to identify recombination within assemblage A one would need to use more variable genetic loci that are conserved in all assemblage A isolates. This type of typing will be possible to develop now when more genomic data exists from the assemblage A group. As the cost of sequencing continues to decrease and *Giardia* genome sequencing can be done without axenization [41] future efforts might target a larger panel of AI and AII genomes in order to exploit the genome-wide diversity rather than a few genes to identify further assemblage substructure. Thus, the level of recombination within and between assemblages but also associations between genotype and symptoms might be possible to resolve in the future. However, already now the combined data suggest that assemblage A and B are actually two different *Giardia* species [9].

For the first time we have identified a set of orthologous genes in assemblage A that show large sequence divergence between different assemblage A isolates (Additional file 6). The majority of genes are hypothetical proteins but some have homology to NADPH oxidoreductases, BPILs and cysteine proteases that have earlier been suggested to be important during host-parasite interactions [25]. Future detailed studies of each protein will show what roles they have during *Giardia* infections. The genes with high levels of sequence divergence can often be found in the variable regions of the *Giardia* genomes containing VSPs and HCMPs and these regions (the *Giardia* variome) make up most of the genetic differences between different *Giardia* isolates. Future studies of the *Giardia* “variome” using long-read sequencing techniques can generate more complete *Giardia* genomes. These variable regions of the *Giardia* genomes can in turn be used in molecular typing, diagnostics and epidemiology, and can reveal molecular signatures, which may relate to infectivity, pathogenesis and drug resistance.

## Conclusions

The genome of *G. intestinalis* is modular with highly conserved core regions mixed up by variable regions containing high levels of SNPs and cysteine rich membrane proteins. Genomes of different assemblage A isolates vary significantly, in terms of gene content, cysteine rich membrane protein repertoire and gene polymorphisms. The large genetic differences within one assemblage of *G. intestinalis* strengthen the argument that the assemblages represent different *Giardia* species.

## Methods

### Ethics

This study was approved by the Regional Committee for Medical Research Ethics at Karolinska Institutet, Sweden (2007/244-31/3) and performed in correspondence to the Declaration of Helsinki. Participation in the study was voluntary and we obtained written informed consent from those participants who were ≥18 years of age at the time of enrollment or their guardians.

### Cell culture

*Giardia* cysts were obtained from a genotyping study at the Karolinska Hospital, the Department of Communicable Disease Control and Prevention, and the Swedish Institute for Communicable Disease Control, in Solna, Sweden [7]. Fecal samples were collected from two patients who were diagnosed with giardiasis, at the Karolinska Hospital, Stockholm, Sweden. Cysts were purified from feces using a sucrose gradient centrifugation as in [42]. Excystation of cysts was done according to Boucher et al. [43]. Recently excysted trophozoites were grown in TYI-S-33 medium [44] containing 10 % fetal bovine serum (GIBCO) or human serum (from the Blood Central, Akademiska Hospital, Uppsala, Sweden) supplemented with 100 μg/ml gentamicin. Cell-growth was estimated as confluence in percent of the area on the bottom of the culture vessel covered with trophozoites every day using a light microscope (Nikon Eclipse, TS 100).

Induction of encystation was achieved through incubation of *Giardia* trophozoites (60–80 % confluence) in encystation medium as in [20]. Trophozoites were incubated for 24 h in encystation medium, tubes containing cysts were centrifuged at 2500 x g for 5 min and the encysting medium was removed. The pellet was re-suspended in 1 ml sterile water and the tubes were stored at 4 °C for 48 h. The cysts were stained with fluorescently labeled monoclonal antibodies (Invitrogen, Cat. No. 73002,) and 4′,6′- diamidino-2-phenyldole (DAPI). One hundred cysts in triplicates, of each population were randomly selected and verified using a fluorescent microscope (AxioPlan 2 imaging, ZEISS). Cysts with an intact cell wall and DAPI stained nuclei were enumerated against cysts lacking DAPI stained nuclei and/or a non-intact or amorphic cyst wall.

### Genotyping of cysts in fecal samples

DNA was extracted using the QIAamp DNA mini kit (Qiagen, Hilden, Germany) according to the manufacturer’s instructions. A disruption of the cysts using a Mini-BeadBeater (Biospec Products Inc., Bartlesville) was performed prior to DNA extraction. The DNA samples were analyzed using a nested β-giardin PCR, a semi-nested gdh PCR and a nested tpi PCR followed by DNA sequencing as in Lebbad et al. [7].

### Chromosome separation using PFGE

Trophozoites of three different isolates (WB, AS98 and AS175) were grown to 100 % confluence in 10 ml NUNC-tubes containing TYI-S-33 medium, collected by centrifugation and pooled. Cells were quantified using a Bürker chamber (Tiefe 0.100 mm, 0.0025 mm^2^) and subsequently resuspended in PBS to a total concentration of 2.5×10^8^ cells. *Giardia* trophozoites and a 1.6 % InCert agarose gel-suspension were equilibrated at 42 °C. Equal amounts of cell suspension and InCert agarose gel (Lonza Cat. No. 50121), were mixed together and cast in sample molds (Bio-Rad Laboratories Cat. No. 277433). The plugs were treated with EPS-buffer (1 % sarcosinate, EDTA, (0.5 M) pH 8.0, proteinase K (2 mg/ml)) and incubated at 42 °C for 72 h. The EPS-buffer was changed every 24 h. PFGE grade agarose (Bio-Rad Laboratories Cat. No. 162–0137) was dissolved in 0.5 × TBE, to a final concentration of 1 %, melted and equilibrated at 60 °C before casting the gel. After polymerization, the DNA-plugs were transferred to the wells and the wells were covered by agarose-solution to achieve a smooth surface of the gel. The pulsed field was performed according to manufacturer’s recommendations for separation of chromosomes in the range of 1–4 Mbp. The smallest chromosomes are shown in Additional file 2.

### Sequencing, read mapping, assembly and annotation

Whole genome shotgun sequencing was performed using the 454 GS Titanium instrument. Preparation and sequencing of the sample was performed according to the manufacturer’s instructions. Base-calling of flowgrams was performed using the bundled 454 software. The sequencer generated 117 and 384 million base pairs respectively for AS98 and AS175, corresponding to 293,759 and 1,139,958 reads (Additional file 3). Genome analysis was performed in two steps, first involving the mapping of sequence reads to the chromosome level assembly of *G. intestinalis* isolate WB (www.Giardiadb.org) using the alignment program ssaha2 [45]. Reads that did not align were subsequently assembled *de novo* using the sequence assembly program MIRA [46] version 3.2.0 with the parameters “--job = denovo,genome,454,normal -SK:mnr = yes 454_SETTINGS -LR:wqf = no -notraceinfo -AS:epoq = no”. This combined genome analysis strategy was used since a reference mapping strategy alone does not capture unique sequences. Reads that aligned to more than one location were removed, since the origin of such reads cannot be determined. Frame shifts were corrected by clustalw alignment to the corresponding gene in isolate WB, using an in-house developed script (available on request).

Gene annotations were transferred from the WB genome using NCBI BLAST and custom Perl scripts, and annotations were manually curated using the visualization program Artemis Comparison Tool [47]. Gene calling was performed on shorter contigs using the program GeneMark [48]. Genes with frame shifts, caused by putative 454 sequencing errors, were not annotated. The coverage distribution was inspected using the assembly visualization software Tablet and custom Perl scripts. Duplicated regions were defined as longer stretches with stable coverage distribution, having sequence reads in different directions with different start and stop positions.

Isolate-specific genes were identified using a combination of automatic searches and manual inspection of results. Statistics was done using the R statistical platform. The genome sequences were sent to NCBI under the accession numbers CAHQ00000000 (AS175) and CVLA01000001-CVLA01000356 (AS98) and they will be searchable at www.GiardiaDB.org together with RNA sequencing data from the AS175 isolate [12].

### Allelic sequence heterozygosity, single nucleotide polymorphisms and evolutionary analysis

Allelic sequence heterozygosity (ASH) and single nucleotide polymorphisms (SNP) were identified from the ssaha2 alignments using custom Perl scripts. Only multiple alignment positions where the sequence coverage exceeded 6× were used in order to avoid low quality regions. Furthermore, positions with more than 50× coverage were ignored because of the risk that these represent collapsed repeats. The genome coverage of the AS98 isolate is in average lower (8X) than the AS175 isolate (20X), which most likely will result in an underestimation of ASH in AS98. Positions containing gaps or undetermined nucleotides were ignored. ASH positions were defined to contain at least 25 % of the second base. The chromosome wide distribution of ASH and SNPs was plotted in sliding windows, with a 5 kb window size and 2.5 kb step size. dN/dS ratios were calculated using the yn00 program [49].

### Phylogenetic analysis of BPIL

The amino acid sequences of all identified BPIL proteins in available *G. intestinalis* genome sequences were aligned with putative *G. muris* BPILs using MAFFT, version 7.215 [50]. 485 unambiguously aligned amino acid positions useful for phylogenetic reconstruction were identified using BMGE [51]. The phylogenetic analysis was performed using RAxML, version 8.1.15 [52] with the LG4X model [53]. The support values from 500 bootstrap replicated were mapped onto the best-scoring ML tree obtained in the same run (option –f a).

### Variant-specific surface proteins

To identify vsp sequences in AS175 and AS98, we ran the emboss program getorf with the parameters “-methionine -minsize 300 -find 1”. Nucleotide sequences were translated into amino acid sequences, and filtered by length, selecting only sequences >400 amino acids. Annotated vsp genes were extracted from the WB genome, and these were used to identify vsp sequences among the extracted ORFs. The NCBI BLASTP program was used to with the WB sequences as a database, and vsp sequences were defined as hits with E-value < 1e–10. A distance matrix was created using the D2S model implemented in the jD2Stat program, and a neighbor-joining phylogenetic tree was inferred with phylip v3.695.

### Epitope tagging of BPIL proteins for localization studies in *Giardia*

The PHA-5 plasmid was used for all the transfections [54]. The plasmid carries the puromycin N-acetyltransferase (PAC) gene, enabling selection by 50 μg/ml of puromycin in *Giardia*. The plasmid contains a 3×HA tag making it possible to epitope tag proteins.. The genes selected for cloning and tagging into the PHA-5 vector were amplified using PCR from genomic DNA of *G. intestinalis* WB-C6 (see Additional file 9 for primer sequences). Transfection of *G. intestinalis* WB trophozoites (approximately 1 × 10^7^ cells) with 20 μg of plasmid DNA was done as in REF. Immunofluorescence was performed as in REF. . Transfected *G. intestinalis* cells were fixed with paraformaldehyde (PFA). 15 μl of anti-HA monoclonal antibody (Alexa Fluor labeled MonoHA) diluted 1:250 times was added and incubated for 2 h at room temperature. The wells were washed 5 times with PBS before addition of 15 μl of the secondary anti-mouse antibody conjugated to Alexa 488 diluted 1:200 times, followed by incubation for 1 h at room temperature. 3 μl of mounting media Vectashield containing DAPI was added before microscopy with a Zeiss Axioplan2 fluorescence microscope. The images were processed using the software Axiovision Rel. 4.8.

## Additional files

Additional file 1:
**Biological characterization of the new assemblage AII isolates.** Trophozoite growth rate in medium supplemented with either bovine or human serum. (DOCX 54 kb) Additional file 2:
**Pulse field analysis of the new assemblage** **A-II** **isolates.** Pulse field analyses of the smallest chromosomes (chromosome 1 and 2) of isolates WB, AS98 and AS175. (EPS 1145 kb) Additional file 3:
**Distribution of read length and assembly positions.** (DOCX 220 kb) Additional file 4:
**Unique assemblage AII genes.** Unique genes in the assemblage AII genomes AS98 and AS175 compared to assemblageAI isolate WB. (XLSX 40 kb) Additional file 5:
**HCMP variability. Variability of HCMP genes in the different assemblage A isolates, WB, AS98 and AS175.** (DOCX 686 kb) Additional file 6:
**Nucleotide diversity between assemblage A orthologs.** List of assemblage AI and AII orthologs and the nucleotide diversity of the alignment. The most diverse genes are at the top, with decreasing diversity. (XLS 690 kb) Additional file 7:
**Strcutural modellling of two** ***Giardia*** **BPI-like** **proteins.** (DOCX 486 kb) Additional file 8:
**Localization of** **BPI-like** **proteins in** ***Giardia*** **using epitope tagging.** (DOCX 244 kb) Additional file 9:
**Primers and cloning of** **BPI-like** **genes.** (DOCX 126 kb)

## Abbreviations

SNP: single nucleotide polymorphisms
ASH: allelic sequence heterozygosity
VSP: variant-specific surface proteins
HCMP: high cysteine membrane proteins
BPIL: Bactericidal/Permeability Increasing-like protein
MLG: multi-locus genotype

## References

[1] Ankarklev J, Jerlstrom-Hultqvist J, Ringqvist E, Troell K, Svard SG (2010). Behind the smile: cell biology and disease mechanisms of Giardia species. Nat Rev Microbiol.

[2] Stark D, Barratt JL, van Hal S, Marriott D, Harkness J (2009). Clinical significance of enteric protozoa in the immunosuppressed human population. Clin Microbiol Rev.

[3] Sprong H, Caccio SM, van der Giessen JW (2009). Identification of zoonotic genotypes of Giardia duodenalis. PLoS Negl Trop Dis.

[4] Caccio SM, Ryan U (2008). Molecular epidemiology of giardiasis. Mol Biochem Parasitol.

[5] Haque R, Roy S, Kabir M, Stroup SE, Mondal D (2005). Giardia assemblage A infection and diarrhea in Bangladesh. J Infect Dis.

[6] Lebbad M, Mattsson JG, Christensson B, Ljungstrom B, Backhans A (2010). From mouse to moose: multilocus genotyping of Giardia isolates from various animal species. Vet Parasitol.

[7] Lebbad M, Petersson I, Karlsson L, Botero-Kleiven S, Andersson JO (2011). Multilocus Genotyping of Human Giardia Isolates Suggests Limited Zoonotic Transmission and Association between Assemblage B and Flatulence in Children. PLoS Negl Trop Dis.

[8] Morrison HG, McArthur AG, Gillin FD, Aley SB, Adam RD (2007). Genomic minimalism in the early diverging intestinal parasite *Giardia lamblia*. Science.

[9] Franzen O, Jerlstrom-Hultqvist J, Castro E, Sherwood E, Ankarklev J (2009). Draft genome sequencing of giardia intestinalis assemblage B isolate GS: is human giardiasis caused by two different species?. PLoS Pathog.

[10] Adam RD, Dahlstrom EW, Martens CA, Bruno DP, Barbian KD (2013). Genome sequencing of Giardia lamblia Genotypes A2 and B isolates (DH and GS) and comparative analysis with the genomes of Genotypes A1 and E (WB and Pig). Genome Biol Evol.

[11] Jerlstrom-Hultqvist J, Franzen O, Ankarklev J, Xu F, Nohynkova E (2010). Genome analysis and comparative genomics of a Giardia intestinalis assemblage E isolate. BMC Genomics.

[12] Franzen O, Jerlstrom-Hultqvist J, Einarsson E, Ankarklev J, Ferella M (2013). Transcriptome profiling of Giardia intestinalis using strand-specific RNA-seq. PLoS Comput Biol.

[13] Tolba ME, Kobayashi S, Imada M, Suzuki Y, Sugano S (2013). Giardia lamblia Transcriptome Analysis Using TSS-Seq and RNA-Seq. PLoS One.

[14] Geurden T, Geldhof P, Levecke B, Martens C, Berkvens D (2008). Mixed Giardia duodenalis assemblage A and E infections in calves. Int J Parasitol.

[15] Adam RD (2000). The Giardia lamblia genome. Int J Parasitol.

[16] Prabhu A, Morrison HG, Martinez CR, Adam RD (2007). Characterisation of the subtelomeric regions of Giardia lamblia genome isolate WBC6. Int J Parasitol.

[17] Adam RD, Nigam A, Seshadri V, Martens CA, Farneth GA (2010). The *Giardia lamblia vsp* gene repertoire: characteristics, genomic organization, and evolution. BMC Genomics.

[18] Chan CX, Bernard G, Poirion O, Hogan JM, Ragan MA (2014). Inferring phylogenies of evolving sequences without multiple sequence alignment. Sci Rep.

[19] Davids BJ, Reiner DS, Birkeland SR, Preheim SP, Cipriano MJ (2006). A new family of giardial cysteine-rich non-VSP protein genes and a novel cyst protein. PLoS ONE.

[20] Bernander R, Palm JE, Svard SG (2001). Genome ploidy in different stages of the Giardia lamblia life cycle. Cell Microbiol.

[21] Adam RD (2001). Biology of Giardia lamblia. Clin Microbiol Rev.

[22] Cooper MA, Adam RD, Worobey M, Sterling CR (2007). Population genetics provides evidence for recombination in Giardia. Curr Biol.

[23] Homan WL, Mank TG (2001). Human giardiasis: genotype linked differences in clinical symptomatology. Int J Parasitol.

[24] Li L, Wang CC (2006). A likely molecular basis of the susceptibility of Giardia lamblia towards oxygen. Mol Microbiol.

[25] Ringqvist E, Avesson L, Soderbom F, Svard SG (2011). Transcriptional changes in Giardia during host-parasite interactions. Int J Parasitol.

[26] Wiesner J, Vilcinskas A (2010). Antimicrobial peptides: the ancient arm of the human immune system. Virulence.

[27] Benere E, Geurden T, Robertson L, Van Assche T, Cos P (2010). Infectivity of Giardia duodenalis Assemblages A and E for the gerbil and axenisation of duodenal trophozoites. Parasitol Int.

[28] Emery SJ, Pascovi D, Lacey E, Haynes PA (2015). The generation gap: Proteome changes and strain variation during encystation in Giardia duodenalis. Mol Biochem Parasitol.

[29] Lujan HD, Mowatt MR, Byrd LG, Nash TE (1996). Cholesterol starvation induces differentiation of the intestinal parasite Giardia lamblia. Proc Natl Acad Sci U S A.

[30] Le Blancq SM, Korman SH, Van der Ploeg LH (1992). Spontaneous chromosome rearrangements in the protozoan Giardia lamblia: estimation of mutation rates. Nucleic Acids Res.

[31] Horn D (2014). Antigenic variation in African trypanosomes. Mol Biochem Parasitol.

[32] Kirkman LA, Deitsch KW (2012). Antigenic variation and the generation of diversity in malaria parasites. Curr Opin Microbiol.

[33] Emery SJ, van Sluyter S, Haynes PA (2014). Proteomic analysis in Giardia duodenalis yields insights into strain virulence and antigenic variation. Proteomics.

[34] Nageshan RK, Roy N, Hehl AB, Tatu U (2011). Post-transcriptional repair of a split heat shock protein 90 gene by mRNA trans-splicing. J Biol Chem.

[35] Andersson JO (2012). Double peaks reveal rare diplomonad sex. Trends Parasitol.

[36] Poxleitner MK, Carpenter ML, Mancuso JJ, Wang CJ, Dawson SC (2008). Evidence for karyogamy and exchange of genetic material in the binucleate intestinal parasite *Giardia intestinalis*. Science.

[37] Logsdon JM (2008). Evolutionary genetics: sex happens in Giardia. Curr Biol.

[38] Malik SB, Pightling AW, Stefaniak LM, Schurko AM, Logsdon JM (2008). An expanded inventory of conserved meiotic genes provides evidence for sex in Trichomonas vaginalis. PLoS One.

[39] Ramesh MA, Malik SB, Logsdon JM (2005). A phylogenomic inventory of meiotic genes; evidence for sex in Giardia and an early eukaryotic origin of meiosis. Curr Biol.

[40] Xu F, Jerlstrom-Hultqvist J, Andersson JO (2012). Genome-wide analyses of recombination suggest that Giardia intestinalis assemblages represent different species. Mol Biol Evol.

[41] Hanevik K, Bakken R, Brattbakk HR, Saghaug CS, Langeland N (2015). Whole genome sequencing of clinical isolates of Giardia lamblia. Clin Microbiol Infect.

[42] Lebbad M, Ankarklev J, Tellez A, Leiva B, Andersson JO (2008). Dominance of Giardia assemblage B in Leon, Nicaragua. Acta Trop.

[43] Boucher SE, Gillin FD (1990). Excystation of *in vitro*-derived Giardia lamblia cysts. Infect Immun.

[44] Keister DB (1983). Axenic culture of Giardia lamblia in TYI-S-33 medium supplemented with bile. Trans R Soc Trop Med Hyg.

[45] Ning Z, Cox AJ, Mullikin JC (2001). SSAHA: a fast search method for large DNA databases. Genome Res.

[46] Chevreux B, Pfisterer T, Drescher B, Driesel AJ, Muller WE (2004). Using the miraEST assembler for reliable and automated mRNA transcript assembly and SNP detection in sequenced ESTs. Genome Res.

[47] Carver TJ, Rutherford KM, Berriman M, Rajandream MA, Barrell BG (2005). ACT: the Artemis Comparison Tool. Bioinformatics.

[48] Besemer J, Lomsadze A, Borodovsky M (2001). GeneMarkS: a self-training method for prediction of gene starts in microbial genomes. Implications for finding sequence motifs in regulatory regions. Nucleic Acids Res.

[49] Yang Z (2007). PAML 4: phylogenetic analysis by maximum likelihood. Mol Biol Evol.

[50] Katoh K, Standley DM (2013). MAFFT multiple sequence alignment software version 7: improvements in performance and usability. Mol Biol Evol.

[51] Criscuolo A, Gribaldo S (2010). BMGE (Block Mapping and Gathering with Entropy): a new software for selection of phylogenetic informative regions from multiple sequence alignments. BMC Evol Biol.

[52] Stamatakis A (2014). RAxML version 8: a tool for phylogenetic analysis and post-analysis of large phylogenies. Bioinformatics.

[53] Le SQ, Dang CC, Gascuel O (2012). Modeling protein evolution with several amino acid replacement matrices depending on site rates. Mol Biol Evol.

[54] Jerlstrom-Hultqvist J, Stadelmann B, Birkestedt S, Hellman U, Svard SG (2012). Plasmid vectors for proteomic analyses in Giardia: purification of virulence factors and analysis of the proteasome. Eukaryot Cell.

