# Supplementary material for: Comparative genomic analyses of freshly isolated Giardia intestinalis assemblage A isolates
Source: BMC Genomics. 2015 Sep 15;16(1):697. doi: 10.1186/s12864-015-1893-6 (PMC4570179; doi:10.1186/s12864-015-1893-6)
Supplement: Additional file 1: — Biological characterization of the new assemblage AII isolates. Trophozoite growth rate in medium supplemented with either bovine or human serum. (DOCX 54 kb) [file 12864_2015_1893_MOESM1_ESM.docx]

**Additional file 1**. The AS 98 and AS 175 isolates were grown in duplicates in either human or bovine serum supplemented growth media. The growth rate was measured in percent confluence using a light microscope every 24 hours up to 96 hours.
