# Supplementary material for: Comparative genomic analyses of freshly isolated Giardia intestinalis assemblage A isolates
Source: BMC Genomics. 2015 Sep 15;16(1):697. doi: 10.1186/s12864-015-1893-6 (PMC4570179; doi:10.1186/s12864-015-1893-6)

**Additional file 3.** A) Distribution of read lengths for AS175 and AS98. Blue line corresponds to AS175 and black line corresponds to AS98. B) Distribution of assembly position with a certain level of coverage. Blue line corresponds to AS175 and black line corresponds to AS98.


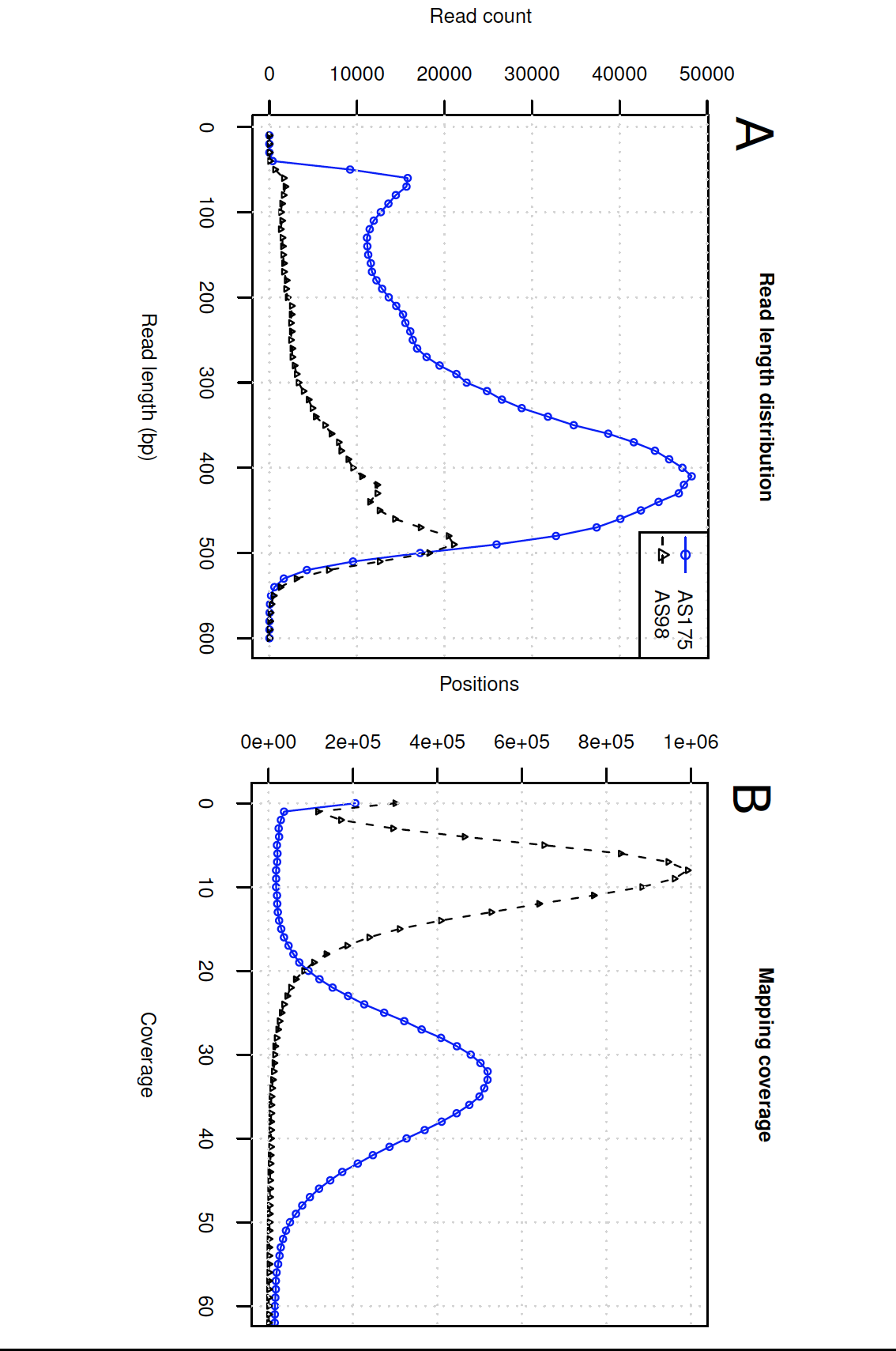

Supplement: Additional file 3: — Distribution of read length and assembly positions. (DOCX 220 kb) [file 12864_2015_1893_MOESM3_ESM.docx]
