# Supplementary material for: Comparative genomic analyses of freshly isolated Giardia intestinalis assemblage A isolates
Source: BMC Genomics. 2015 Sep 15;16(1):697. doi: 10.1186/s12864-015-1893-6 (PMC4570179; doi:10.1186/s12864-015-1893-6)

**Additional file 5.** Variability of HCMP genes in the different assemblage A isolates. Blue: WB, Green: AS98, Red: AS175. The size of the HCMP family was estimated using a profile hidden markov model constructed from the HCMP genes from the WB genome using HMMER version 3.0 [[47](#_ENREF_47)]. The combined alignment length of sequence reads was calculated and divided with the average assembly coverage, which represents a rough estimation of the HCMP family size in each AII isolate.


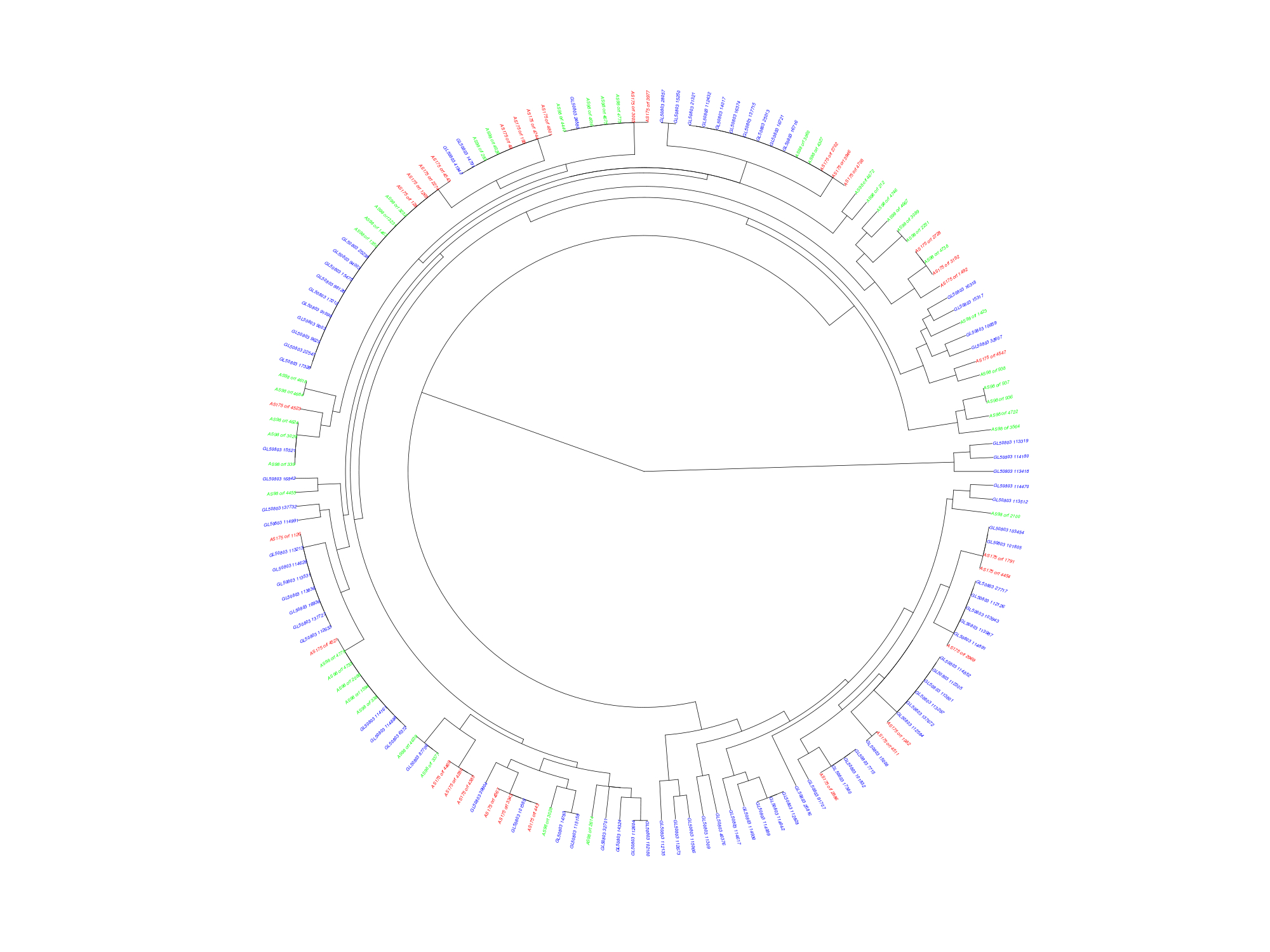

Supplement: Additional file 5: — HCMP variability. Variability of HCMP genes in the different assemblage A isolates, WB, AS98 and AS175. (DOCX 686 kb) [file 12864_2015_1893_MOESM5_ESM.docx]
