# Supplementary material for: Comparative genomic analyses of freshly isolated Giardia intestinalis assemblage A isolates
Source: BMC Genomics. 2015 Sep 15;16(1):697. doi: 10.1186/s12864-015-1893-6 (PMC4570179; doi:10.1186/s12864-015-1893-6)
Supplement: Additional file 7: — Strcutural modellling of two Giardia BPI-like proteins. (DOCX 486 kb) [file 12864_2015_1893_MOESM7_ESM.docx]

**Additional file 7. Structure prediction of *Giardia* BPILs.**


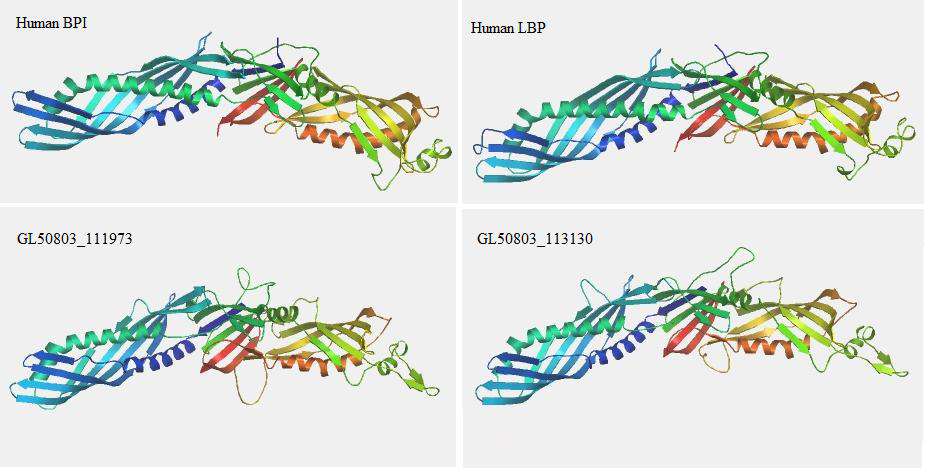


Structure prediction of human BPI (PDB ID: 1BP1, Chain: A), human LBP **(**Swiss**-**

Prot ID: P18428) and the two BPI-like proteins GL50803_113130 and GL50803_111973 in the *G. intestinalis* WB isolate. Note that the N-terminal of the proteins is colored blue while the C-terminal is colored yellow-orange. For the protein GL50803_111973 the sequence identity with the human BPI is 13.737% and for the GL50803_113130 is 12.727%.
