# Supplementary figures and images for: Comparative genomic analyses of freshly isolated Giardia intestinalis assemblage A isolates
Source: BMC Genomics. 2015 Sep 15;16(1):697. doi: 10.1186/s12864-015-1893-6 (PMC4570179; doi:10.1186/s12864-015-1893-6)

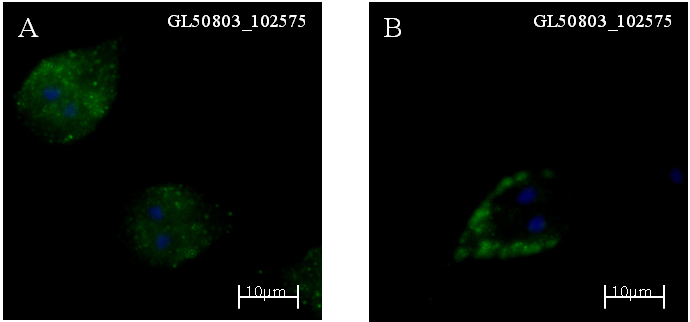

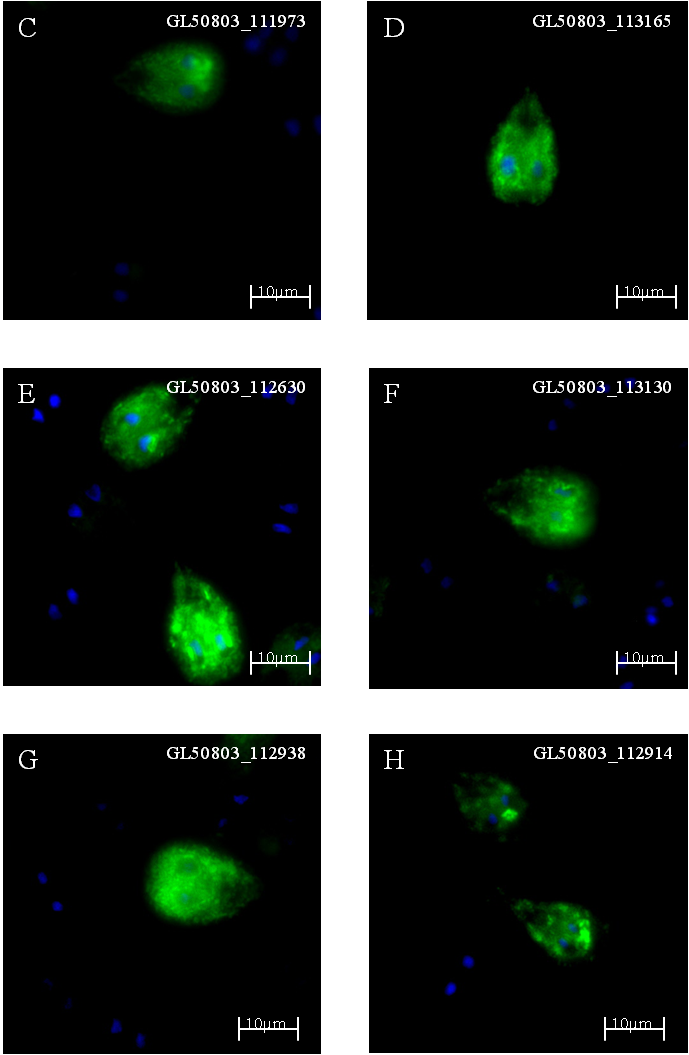

Supplement: Additional file 8: — Localization of BPI-like proteins in Giardia using epitope tagging. (DOCX 244 kb) [file 12864_2015_1893_MOESM8_ESM.docx]
