# Supplementary material for: Comparative genomic analyses of freshly isolated Giardia intestinalis assemblage A isolates
Source: BMC Genomics. 2015 Sep 15;16(1):697. doi: 10.1186/s12864-015-1893-6 (PMC4570179; doi:10.1186/s12864-015-1893-6)
Supplement: Additional file 9: — Primers and cloning of BPI-like genes. (DOCX 126 kb) [file 12864_2015_1893_MOESM9_ESM.docx]

**List primers used for cloning of BPIL proteins from *Giardia.***

| **Name of Primer** | **Direction** | **Primer Sequence** |
| --- | --- | --- |
|  |  |  |
| pPAC-GL50803_16293-3xHA-C frwd | Forward | TCTAGATCTGGAGGAGCTGTCCTCTGGCG |
| pPAC-GL50803_16293-3xHA-C rev | Reverse | GCGGCCGCCAGGAAAAGTACGGATTACCCGCTATGTCAGCC |
|  |  |  |
| pPAC-GL50803_102575-3xHA-C frwd | Forward | ACGCGTAATAATATGGTAAAAAGATACACAGC |
| pPAC-GL50803_102575-3xHA-C rev | Reverse | GCGGCCGCCAAGTGAAGTACTGATTCTTCTCA |
|  |  |  |
| pPAC-GL50803_112938-3xHA-C frwd | Forward | ACGCGTCCTCATGACAGTGAAACAGAG |
| pPAC-GL50803_112938-3xHA-C rev | Reverse | GCGGCCGCCAAGCAAAGTACTGGTTTTTCT |
|  |  |  |
| pPAC-GL50803_112914-3xHA-C frwd | Forward | ACGCGTCCTCATGACAGTGAAACAGAG |
| pPAC-GL50803_112914-3xHA-C rev | Reverse | GCGGCCGCCAAGCAAAGTACTGGTTCTTCT |
|  |  |  |
| pPAC-GL50803_112630-3xHA-C frwd | Forward | ACGCGTTACTAAATTGGGCTCGACAA |
| pPAC-GL50803_112630-3xHA-C rev | Reverse | GCGGCCGCGAAAAATATCGATTCTTCTCGA |
|  |  |  |
| pPAC-GL50803_113165-3xHA-C frwd | Forward | ACGCGTAGTAAGTTAAAATACGTCCATTTA |
| pPAC-GL50803_113165-3xHA-C rev | Reverse | GCGGCCGCCAAGCGAAGTATTGGTTCTT |
|  |  |  |
| pPAC-GL50803_111973-3xHA-C frwd | Forward | ACGCGTGCCTATTTAAGCTTTTAGTGAAT |
| pPAC-GL50803_111973-3xHA-C rev | Reverse | GCGGCCGCCAGGCAAAGTAGTGATTCTTCT |
|  |  |  |
| pPAC-GL50803_113130-3xHA-C frwd | Forward | ACGCGTCAGAATGAGCGTAAGAGGTATGC |
| pPAC-GL50803_113130-3xHA-C rev | Reverse | GCGGCCGCGCAAAATACCGATTCTTCTCGA |

**List of all restriction sites used for the cloning of the BPI-like proteins from *Giardia.***

| **Gene** | **Restriction site 1** | **Restriction site 2** |
| --- | --- | --- |
| GL50803_16293 | XbaI | NotI |
| GL50803_102575 | MluI | NotI |
| GL50803_112938 | MluI | NotI |
| GL50803_112914 | MluI | NotI |
| GL50803_112630 | MluI | NotI |
| GL50803_113165 | MluI | NotI |
| GL50803_11973 | MluI | NotI |
| GL50803_113130 | MluI | NotI |
